# Increasing of Blood-Brain Tumor Barrier Permeability through Transcellular and Paracellular Pathways by Microbubble-Enhanced Diagnostic Ultrasound in a C6 Glioma Model

**DOI:** 10.3389/fnins.2017.00086

**Published:** 2017-02-23

**Authors:** Jinlong Zhang, Heng Liu, Xuesong Du, Yu Guo, Xiao Chen, Shunan Wang, Jingqin Fang, Peng Cao, Bo Zhang, Zheng Liu, Weiguo Zhang

**Affiliations:** ^1^Department of Radiology, Research Institute of Surgery, Daping Hospital, Third Military Medical UniversityChongqing, China; ^2^GE HealthCareShanghai, China; ^3^Four and the State Key Laboratory of Trauma, Burns and Combined Injury, Research Institute of Surgery, Daping Hospital, Third Military Medical UniversityChongqing, China; ^4^Department of Ultrasound, Xinqiao Hospital, Third Military Medical UniversityChongqing, China; ^5^Chongqing Clinical Research Center for Imaging and Nuclear MedicineChongqing, China

**Keywords:** glioma, blood-brain tumor barrier, permeability, diagnostic ultrasound, dynamic contrast-enhanced-MRI

## Abstract

Most of the anticancer agents cannot be efficiently delivered into the brain tumor because of the existence of blood-brain tumor barrier (BTB). The objective of this study was to explore the effect of microbubble-enhanced diagnostic ultrasound (MEUS) on the BTB permeability and the possible mechanism. Glioma-bearing rats were randomized into three groups as follows: the microbubble-enhanced continued diagnostic ultrasound (MECUS) group; the microbubble-enhanced intermittent diagnostic ultrasound (MEIUS) group and the control group. The gliomas were insonicated through the skull with a diagnostic ultrasound and injected with microbubbles through the tail veins. Evans Blue (EB) and dynamic contrast-enhanced-MRI were used to test changes in the BTB permeability. Confocal laser scanning microscopy was used to observe the deposition of the EB in the tumor tissues. The distribution and expression of junctional adhesion molecule-A (JAM-A) and calcium-activated potassium channels (*K*_Ca_ channels) were detected by a Western blot, qRT-PCR, and immunohistochemical staining. In the MEUS groups, the EB extravasation (11.0 ± 2.2 μg/g in MECUS group and 17.9 ± 2.3 μg/g in MEIUS group) exhibited a significant increase compared with the control group (5.3 ± 0.9 μg/g). The MEIUS group had more EB extravasation than the MECUS group. The *K*_trans_ value of the dynamic contrast-enhanced-MRI in the MEUS groups was higher than that of the control group and correlated strongly with the EB extravasation in the tumor (*R*^2^ = 0.97). This showed that the *K*_trans_ value might be a non-invasive method to evaluate the BTB permeability in rat glioma after microbubble-enhanced ultrasound treatment.Western blot, qRT-PCR and immunohistochemical staining revealed that MEUS increased the *K*_Ca_ channels expression and reduced JAM-A expression in glioma. This change was more obvious in the MEIUS group than in the MECUS group. The results demonstrated that MEUS effectively increased the BTB permeability in glioma. The mechanisms might involve the up-regulation of *K*_Ca_ channels expression and affecting the formation of tight junctions in the BTB by a reduction of JAM-A expression. These findings might provide some new guidance for glioma drug therapy.

## Introduction

Blood-brain barrier (BBB) is a major limitation to the use of drugs in the brain. Some studies show that nearly 100% of macromolecular and 98% of micromolecular drugs cannot pass through the BBB (Pardridge, 2007). This limitation affects the development of effective drugs for many brain diseases. Studies show that the glioma vasculature permeability is heterogeneous, and there are barriers to drug therapy, such as edema and increased interstitial pressures (Fukumura and Jain, 2007). Furthermore, although the blood vessels in glioma often do not have a fully intact BBB and are somewhat permeable, the feeding capillaries reserve the characteristics of the BBB and form a blood-brain tumor barrier (BTB, Hu et al., 2007; Eichler et al., 2011). BTB is similar to BBB, which is located between tumor cells and microvessels formed by highly specialized endothelial cells. Although the permeability of BTB is higher than that of BBB (Yuan et al., 1994), the existence of a BTB obviously limits the delivery of drugs into glioma. Techniques that can help drugs pass across the BTB could enable the use of many anti-tumor agents in glioma therapy.

Previous studies have demonstrated that microbubbles increase the possibility of acoustic cavitation and cavitation-related vascular damage effects, such as microvascular rupture and petechial hemorrhage (Miller and Gies, 2000a; Miller and Quddus, 2000b; Miller, 2007), in which microbubbles served as cavitation nuclei to induce cavitation (Miller and Thomas, 1995). These vascular effects could be therapeutically useful in thrombolysis and gene or drug delivery (Molina et al., 2006; Ferrara et al., 2007; Xie et al., 2009; Kutty et al., 2014; Sato et al., 2015; Burgess and Hynynen, 2016). Recently, some studies showed that the permeability of the BTB is increased by focused ultrasound and low-frequency ultrasound in the presence of an ultrasound contrast agent (Liu et al., 2006; Hynynen, 2008; Xia et al., 2012; Diaz et al., 2014; Aryal et al., 2015). This technique non-invasively enhances the uptake of drugs or genes to the targeted glioma tissues (Diaz et al., 2014; Aryal et al., 2015). The above effect is reversible, allowing for a time window for drug delivery to the target site of the brain tumor. However, the potential molecular mechanism involved in this process still need to be investigated.

In general, there are two pathways for drugs to cross the BTB: transcellular pathway and paracellular pathway. The paracellular pathway is often regarded as the main path for the absorption of hydrophilic drugs (peptides, proteins, etc.; (Salama et al., 2006)). Ningaraj et al. showed that *K*_Ca_ channels played an important role in the transcellular pathway regulation of BTB permeability. These channels were effective targets for regulating the formation of transport vesicles in both the glioma capillary endothelium and tumor cells (Ningaraj et al., 2003). And there is no report about the changes of calcium-activated potassium channels expression following ultrasound irradiation and microbubbles.

The BBB has complex tight junctions and an inactive endocytic transport (Jolliet-Riant and Tillenment, 1999). Integral tight junction proteins are claudins, occluding, and junctional adhesion molecules (JAMs). JAM-A is implicated in a variety of physiologic and pathologic processes involving cellular adhesion, such as tight junction assembly (Yeung et al., 2008). Several studies report that ultrasound irradiation and microbubbles can open the BBB and decrease the expression of junctional proteins, such as claudin-5 and occludin (Sheikov et al., 2008; Xia et al., 2012). However, little is known about the changes of JAM-A expression following the use of ultrasound irradiation and microbubbles.

Therefore, in this study, we applied low-frequency diagnostic ultrasound with microbubbles to increase the BTB permeability in a rat C6 glioma model without damaging the brain tissue. Then, we investigated the effects of MEUS on the distribution and expression levels of the *K*_Ca_ channels and JAM-A to elucidate the possible mechanism involved in this process.

## Materials and methods

### Cell culture

C6 glioma cells were obtained from the cell bank of the Chinese Academy of Sciences (Shanghai, China). The cells were incubated in DMEM/F_12_ medium (Gibco, Carlsbad, CA) supplemented with 10% FBS and 100 units/ml penicillin (HyClone, Logan, AR) at 37°C in a humidified atmosphere containing 95% air and 5% CO_2_.

### Animal models

Ninety healthy adult male Sprague-Dawley rats weighting 230–280 g were anesthetized and placed in a stereotaxic frame (RWD Life Science, Shenzhen, China). Their uppermost cranial hair was removed with the aid of clippers, whereby a midline incision exposed the cranium. A burr hole was drilled in the skull 1 mm anterior to the bregma and 3 mm lateral to the midline. The microsyringe needle advanced to the depth of 5 mm and 1 × 10^6^ C6 glioma cells, resuspended in 10 μL phosphate-buffered saline (PBS), were injected to establish the *in situ* brain glioma model. Eight days after the glioma cell implantation, the rats were prepared for the experiments.

The glioma-bearing rats were randomized into the control group (*n* = 30), the microbubble-enhanced continued diagnostic ultrasound (MECUS) group (*n* = 30), and the microbubble-enhanced intermittent diagnostic ultrasound (MEIUS) group (*n* = 30).

### Ethics statement

All the procedures were performed in accordance with the approval of the Institutional Animal Care and Use Committee of the Third Military Medical University, Daping Hospital. All the animals received care in accordance with the Guide for the Care and Use of Laboratory Animals. At the end of the experiment, the animals were euthanized using an overdose of 10% chloral hydrate.

### Microbubble

Zhifuxian (Liu et al., 2011; Wu et al., 2014), a lipid-coated microbubble, was used for the nucleation of acoustic cavitation. It was prepared by lyophilization of two lipids suspension, 1,2-Dipalmitoyl-sn-glycero-3-phosphoglycerol (DPPG) and 1,2-Distearoyl-sn–glycero-3-phosphoethanolamine (DSPE), and agitating with perfluoropropane gas using a high-speed mechanical amalgamator. The size distribution and concentration of the microbubbles were determined by an RC-3000 Resistance Particle Counter (OMEC Technology Co., Ltd. China). The microbubbles had a mean particle diameter of 2 μm, with 98% of the particles less than 8 μm and a bubble concentration of 9 × 10^10^/mL.

### Treatment protocols

All the tumor-bearing rats were anesthetized with 10% chloral hydrate. The rats were fixed on the experimental table. Their uppermost cranial hair was removed by 8% Na_2_S. An S5-1 transducer (IE33; Philips ultrasound, Bothell WA, USA) with a frequency of 1.7 MHz was placed above the temporal bone (with a depth of 5 cm), and the targeted tumor area was kept at the ultrasound focal point by measuring the distance between the tumor core and the temporal bone skin through an MRI examination. Ultrasound gel was administered between the transducer and the temporal bone. In the MECUS group, the rats were directly insonated using a continued ultrasound.The injected dose of microbubbles through the tail vein was 1.5 mL/kg. The duration of the ultrasound sonication was 10 min, and the mechanical index of the ultrasound was 1.3. Continued ultrasound irradiation was replaced by intermittent ultrasound (2-2s) irradiation in the MEIUS group. While in the control group, the microbubbles were replaced by 1.5 mL/kg saline and a sham ustrasound insonation was applied instead of the ultrasound irradiation.

### Measurement of the BTB permeability by DCE-MRI

All the rats underwent an MRI with a Bruker BioSpec 7T/20 cm system (Bruker, Ettlingen, Germany) using a head surface coil before ultrasound irradiation. The sequences used in this study were as follows: T_2_-weighted image (T_2_WI; repetition time = 2500 ms, echo time = 45 ms, field of view = 35 mm × 35 mm, slice thickness = 0.5 mm, NEX = 4, flip angle = 90°).

One hour after the treatment, the rats (*n* = 6 for each group) were subjected to the DCE-MRI to evaluate the increase BTB permeability. To generate the T1 maps from the precontrast images, the DCE FLASH images, with multiple flip angles of 5, 10, 15, and 20°, were acquired. The following parameters were used to acquire the DCE FLASH images: TR = 52.823 ms; TE = 1.765 ms using a 128 × 128 matrix; FOV = 35 mm × 35 mm; and NEX = 1. The effective slice thickness was 0.5 mm. To obtain the dynamic postcontrast DCE FLASH images, a fixed flip angle of 15° was used, with 6.761 s for 540.907 s (80 time points). Acquisition of the DCE FLASH images was started before the administration of the contrast to obtain the baseline T_1_ signals. When the third dynamic loop finished, 0.5 mmol/ mL of Omniscan (GE Healthcare, Cork, Ireland) was administered at a dosage of 0.1 mmol/kg body weight by hand push within 4 s. All the images were transferred to an independent workstation for quantitative analysis by a non-commercial software (OmniKinetics, GE Healthcare, China). The reference region model was used to calculate the forward transfer constant *K*_trans_.

### Measurement of the BTB permeability by evans blue (EB)

Two percent EB (50 mg/kg) was injected into each group of rats through the tail vein before the microbubble injection 24 h later after dynamic contrast-enhancd (DCE)-MRI scanning when treatment was performed again. Then, rats were sacrificed using an overdose of chloral hydrate 1 h after the treatment. The rats were then infused with 0.9% heparinized saline in the left ventricle until a colorless perfusion fluid was obtained from the right atrium. Then, each tumor was cut into two halves: one for the quantitative analysis of EB accumulation and the other for the confocal laser scanning microscopy (CLSM) examination. For the CLSM examination, the tumor parts were extracted for frozen sectioning and cut into 5-mm slices at intervals of 0.8 mm immediately after the heparinized saline infusion. The frozen sections were stained in 5 mg/mL of DAPI (4′,6-diamidino-2-phenylindole) dihydrochloride (Sigma, St. Louis, MO, USA) for 3 min to mark the nuclei, followed by staining with an anti-fade mounting medium (Beyotime, Shanghai, China). The images were recorded using CLSM (TCS SP5; Leica, Wetzlar, Germany). The EB and DAPI dihydrochloride were excited at 620 nm and 454 nm. For the quantitative analysis of EB, the tumor parts were weighed and put into formamide (1 mL/100 mg) at 60°C for 24 h. The concentration of the dye extracted from each brain was determined by a spectrophotometer at 620 nm and was compared to a standard graph created through the recording of the optical densities from serial dilutions of EB in 0.9% sodium chloride solution.

### Immunohistochemistry

The rats (*n* = 5 for each group) were sacrificed by an overdose of chloral hydrate 1 h after the treatment. The brains were extracted from the skull after a perfusion with 200 mL of heparinized saline and then 200 mL of 4% paraformaldehyde. Then, the brains were place in fixative for 1 week and were then immersed in a 30% sucrose solution with PBS for 4 days. Coronal sections were serially cut into pieces at a thickness of 6 μm and at intervals of 0.8 mm on a cryostat. Before the immuno-staining, they were rehydrated in PBS. Then, they were incubated with a 0.3% H_2_O_2_/methonal solution for 10 min at room temperature so that the endogenous peroxidase activity was inactivated. The next step was to wash and block the sections using normal goat serum. Then, the sections were incubated with a rabbit polyclonal anti-JAM-A antibody (Abcam, Cambridge, UK) and a mouse monoclonal anti-α-subunit of *K*_Ca_ channels antibody (Abcam, Cambridge, UK) at 4°C overnight. Negative controls were obtained by using PBS instead of the primary antibody. The other procedures were performed according to immunohistochemistry standard procedures.

### Quantitative real time-polymerase chain reaction (qRT-PCR)

The rats (*n* = 5 for each group) were sacrificed by an overdose of chloral hydrate 1 h after the treatment and the tumor tissues were obtained. The tissues were immediately frozen in liquid nitrogen and stored at −80°C. For qRT-PCR analysis of JAM-A and α-subunit of *K*_Ca_ channels mRNA, total RNA was extracted from tumor tissue using TrizolReagent. RNA samples were subjected to qRT-PCR. Primers used for PCR amplification were as follows: JAM-A (sense: 5′- CGGGACCCCTTGCTTTTATTC- 3′, anti-sense: 5′- GAATGGGGAATGAAAACTTTGTAAC -3′), α-subunit of *K*_Ca_ channels (sense: 5′- CCGCTTTCTTCTGTCTCTGTTAATG - 3′, anti-sense: 5′- TCGGGGATGTGTTGGGTGAG -3′), β-actin (sense: 5′-GTGGGGCCCCAGGCACCA-3′, anti-sense: 5′-GCTCGGCCGTGGTGGTGAAGC-3′). The amplification cycling conditions were as follows: 95°C for 10 min, followed by 40 cycles at 95°C for 10 s, 60°C for 30 s and 72°C for 20 s. The relative JAM-A and α-subunit of *K*_Ca_ channels mRNA levels were calculated as 2^∧−ΔΔ^Ct, where Ct represents the threshold cycle. β-actin was used as the internal reference.

### Western blot assessment

The tumor tissue (*n* = 5 for each group) was obtained after infusing heparinized saline into the blood system. The proteins were extracted, and a Western blot analysis was performed as previously reported (Fang et al., 2013). All of the samples were manipulated in equal conditions. Briefly, a 10% SDS-PAGE was used to separate the protein samples (20 mg), which were transferred to a polyvinylidene difluoride membrane, probed with a rabbit polyclonal anti-JAM-A antibody (diluted 1:2000, Millipore, CA, USA), a mouse polyclonal anti-α-subunit of *K*ca channels antibody (diluted 1:800, Millipore, CA, USA) and a mouse polyclonal antibody β-actin (diluted 1:3000, Santa Cruz Biotechnology Inc., Santa Cruz, CA) overnight at 4°C. After an incubation with secondary antibodies, the protein expressions were detected by enhanced chemiluminescence and were quantified using a Quantity One Software (Bio-Rad, Hercules, CA).

### Histological examination

The rats (*n* = 9 for each group) were sacrificed by an overdose of chloral hydrate 1 h after the treatment. The brains were extracted from the skull after perfusion with 200 mL of heparinized saline and then 200 mL of 4% paraformaldehyde. Then, the brains were placed into a fixative for 1 week and were then immersed in a 30% sucrose solution with PBS for 4 days. Coronal sections were serially cut into pieces at a thickness of 6 μm on a cryostat. Every 30th section was stained with hematoxylin and eosin and TUNEL for histological examination. TUNEL staining was done with an apoptosis detection kit (Roche, USA) according to the manufacturer's instructions. Tumor regions were used as a positive control in experiments. The sections were visualized by two independent blinded observers.

### Statistical analysis

All the data are expressed as the mean ± standard deviation values. A one-way analysis of variance was utilized to determine the significant difference between multiple groups. A *p* < 0.05 was deemed statistically different. All the data were analyzed using SPSS 18.0 software.

## Results

### Measurement of the BTB permeability by EB

**Figure 1:**
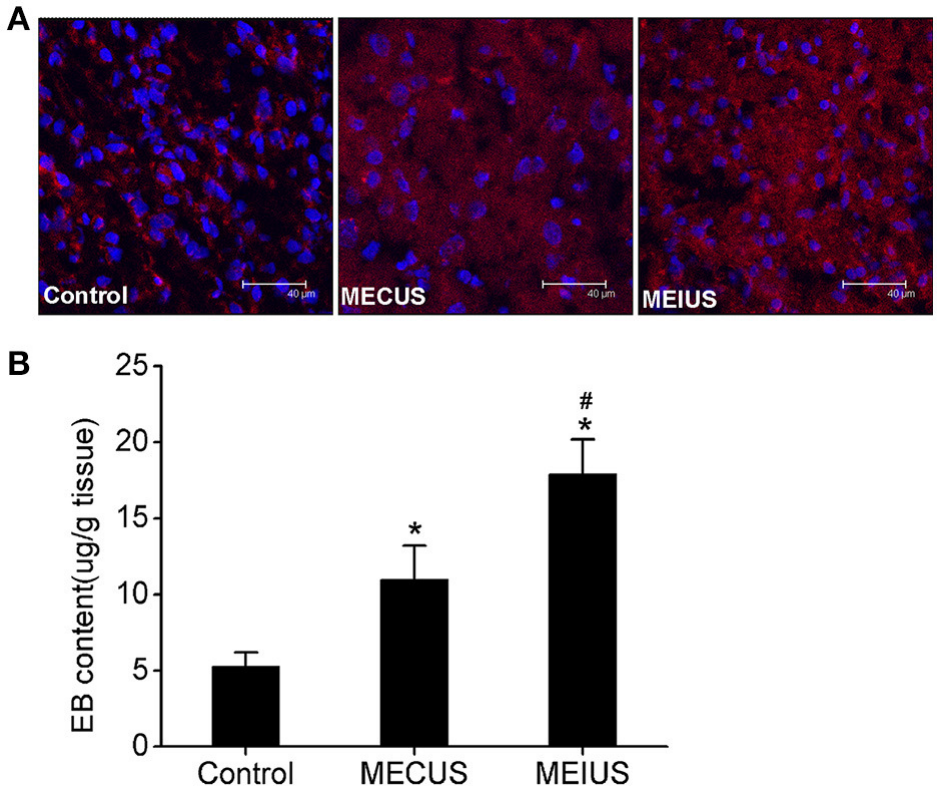
**Quantitative determination the EB extravasation in glioma. (A)** Confocal laser scanning microscopy images depicting the EB deposition in glioma. The red fluorescence represents EB, and the blue fluorescence represents the cell nuclei. The images show that the red fluorescence is distributed in the tumor interstitium in all the groups. The red fluorescence in the microbubble-enhanced ultrasound groups exhibited a significant increase compared with the control group. The MEIUS group had a brighter red fluorescence in the tumor interstitium than the MECUS group. **(B)** EB extravasation (mean ± standard deviation) in the tumor tissues from three different experimental groups (*n* = 6 for each group). ^*^Indicates a significant difference compared with the control group (*p* < 0.05). ^#^Indicates a significant difference compared with the microbubble-enhanced continued diagnostic ultrasound (MECUS) group (*p* < 0.05).

In the MEUS groups, the EB extravasation (11.0 ± 2.2 μg/g in MECUS group and 17.9 ± 2.3 μg/g in MEIUS group) exhibited a significant increase compared with the control group (5.3 ± 0.9 μg/g). The MEIUS group had more EB extravasation than the MECUS group (*p* < 0.05) (Figure 1B). This result showed that the blood vessels in glioma do not have a fully intact BBB, and MEUS increased the BTB permeability. Furthermore, MEIUS more obviously enhanced the BTB permeability than MECUS. CLSM images showed similar results. There was deposition of EB in tumor interstitium in all groups. In the MEUS groups, the deposition of EB in the tumor interstitium exhibited a significant increase compared with the control group. The MEIUS group had a brighter red fluorescence than the MECUS group (Figure 1A). The result showed that MEUS increased the BTB permeability, and this was more obviously exhibited in MEIUS than in MECUS.

### Measurement of the BTB permeability by DCE-MRI

**Figure 2:**
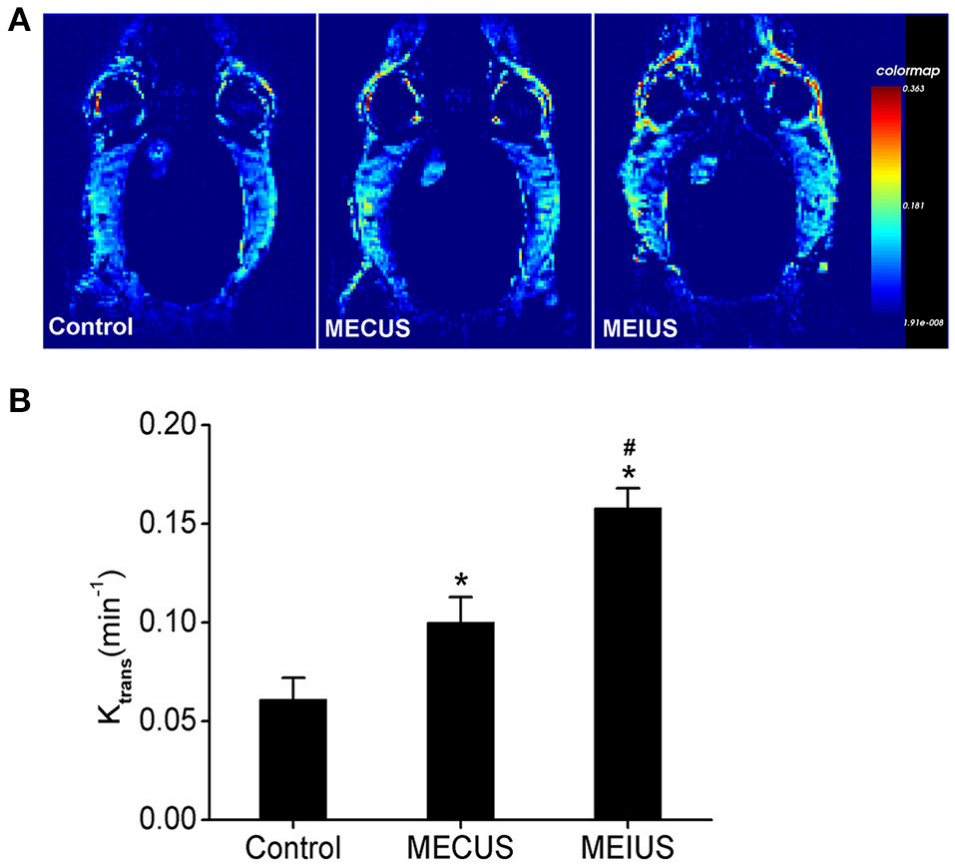
**The ***K***_**trans**_ changes in the tumor tissues from the different groups. (A)** Representative *K*_trans_ maps of rats with glioma in the different groups. **(B)** Changes in the *K*_trans_ value in the different groups were analyzed (*n* = 6 for each group). The values represent the mean ± standard deviation. ^*^Indicates a significant difference compared with the control group (*p* < 0.05). ^#^Indicates a significant difference compared with the MECUS group (*p* < 0.05).

In the MEUS groups, the *K*_trans_ value (0.1 ± 0.013 min^−1^ in MECUS group and 0.158 ± 0.01 min^−1^ in MEIUS group) was significantly increased compared with the control group (0.061 ± 0.011 min^−1^). The MEIUS group had a higher *K*_trans_ value than the MECUS group (*p* < 0.05) (Figure 2B). The result demonstrated that MEUS increased the BTB permeability, and this was exhibited more obviously in MEIUS than in MECUS. The *K*_trans_ map images showed similar results. In the MEUS groups, the tumor color of the *K*_trans_ map images was brighter compared with the control group. The MEIUS group exhibited this more obviously than the MECUS group (Figure 2A). However, there were no obvious changes as tumor tissue in the surrounding brain tissue after MEUS in the *K*_trans_ maps. These results demonstrated that MEUS increased the BTB permeability, and MEIUS exhibited this more obviously than MECUS.

**Figure 3:**
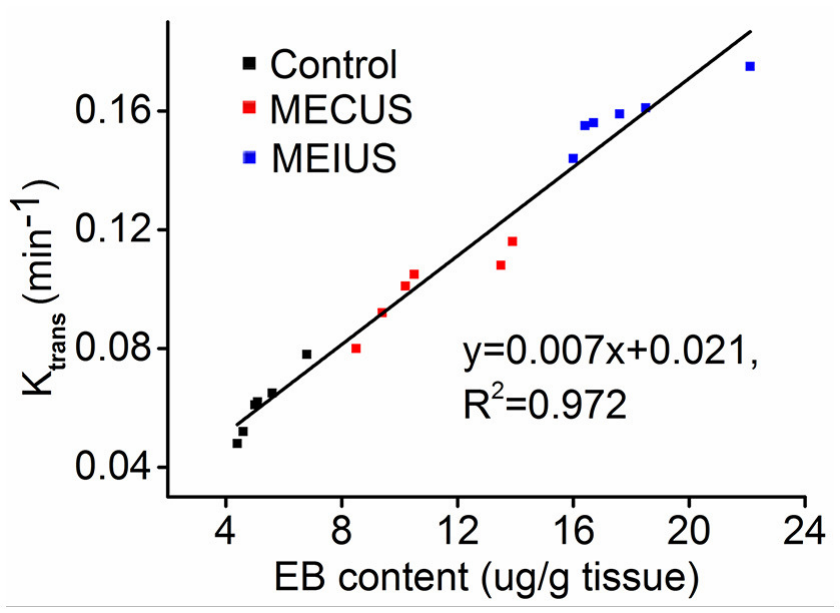
**Scatter plots and fitted linear correlation lines of the EB content versus the ***K***_**trans**_ value in the tumor tissues**. This figure shows that the *K*_trans_ value correlates strongly with the EB extravasation in the tumor tissue (*R*^2^ = 0.97).

Furthermore, we found the *K*_trans_ value correlated strongly with the EB extravasation in the tumor tissue (*R*^2^ = 0.97) (Figure 3). This demonstrated that the *K*_trans_ value could be used as a non-invasive method to evaluate BTB permeability in rat glioma after a microbubble-enhanced ultrasound treatment.

### Distribution and expression changes of JAM-A and the α-subunit of the *K*_Ca_ channels

**Figure 4:**
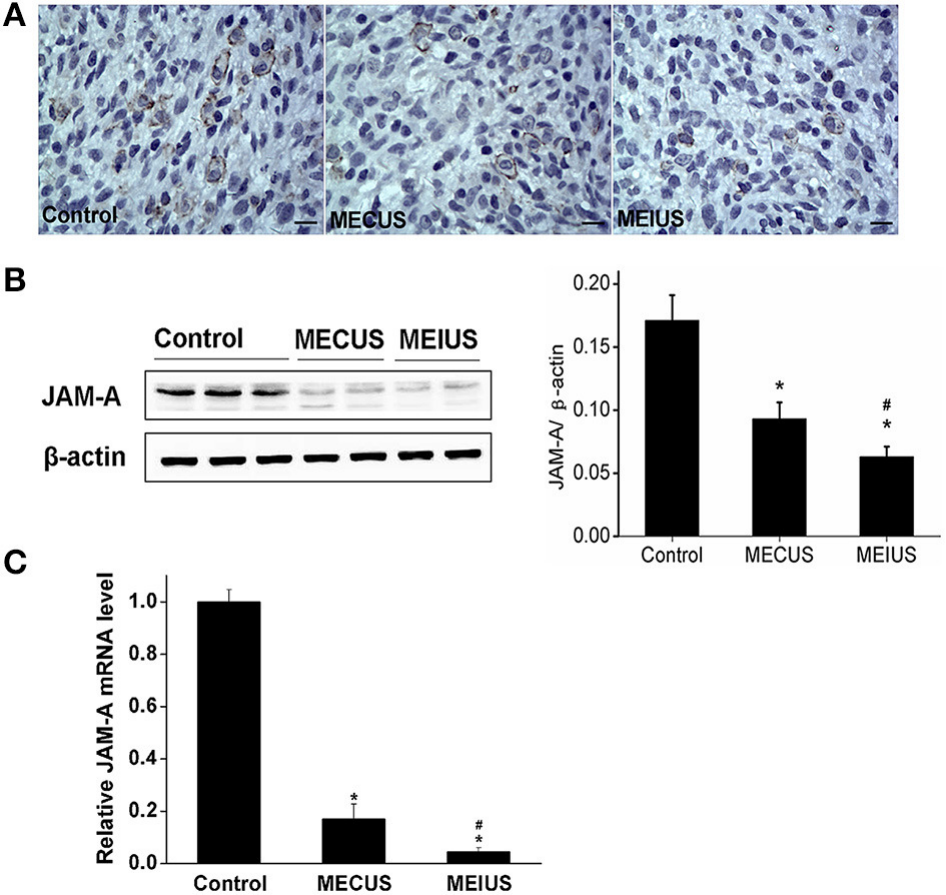
**Distribution and expression changes of junctional adhesion molecule-A (JAM-A) in the tumor tissues from the different groups (scale bar = 20 um). (A)** Distribution and expression of JAM-A by immunohistochemistry. **(B)** The JAM-A protein expression changes by a Western blot analysis in the tumors from the different groups. The result from each group was normalized to β-actin. The quantification of JAM-A expression was performed by scanning the intensity of the densitometry value (*n* = 5 for each group; values represent the mean ± standard deviation). **(C)** The JAM-A mRNA expression changes by a qRT-PCR analysis in the tumors from the different groups (*n* = 5 for each group; values represent the mean ± standard deviation). ^*^Indicates a significant difference compared with the control group (*p* < 0.05). ^#^Indicates a significant difference compared with MECUS group (*p* < 0.05).

**Figure 5:**
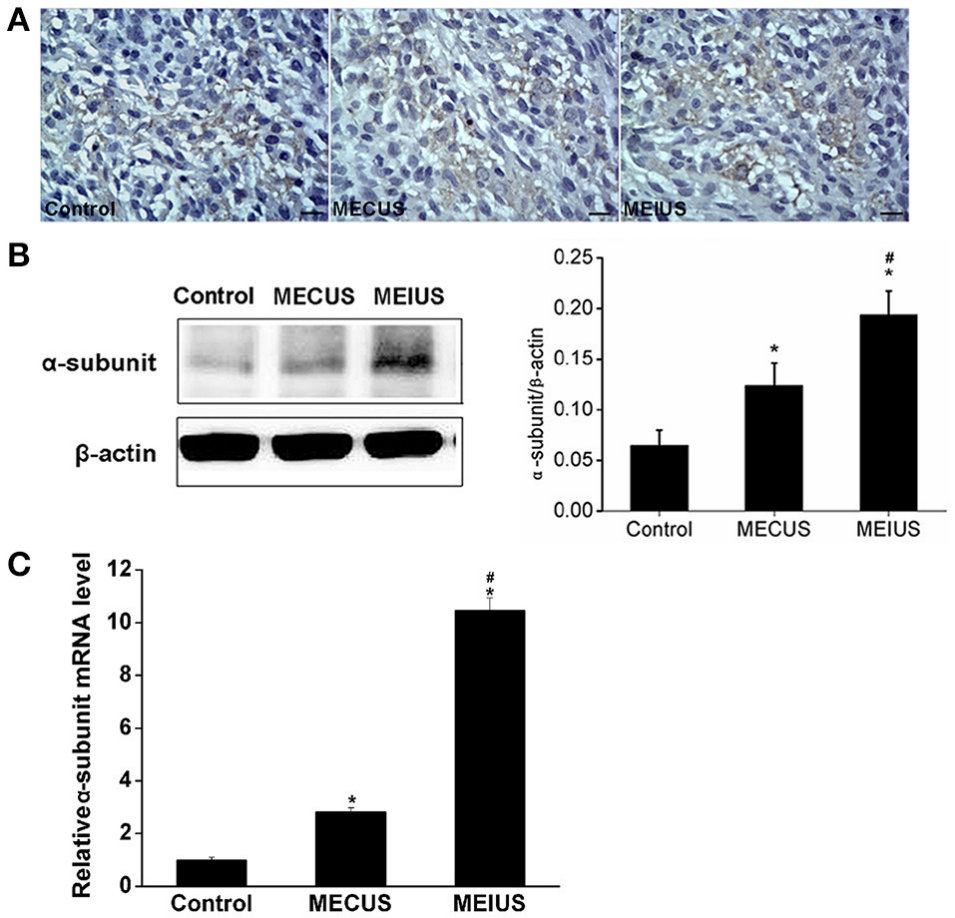
**Distribution and expression changes of the α-subunit of the calcium-activated potassium channels (***K***_**Ca**_ channels) in the tumor tissues from the different groups (scale bar = 20 um). (A)** Distribution and expression of the α-subunit of the *K*_Ca_ channels by immunohistochemistry. **(B)** The α-subunit of the *K*_Ca_ channels protein expression changes by a Western blot analysis in the tumors from the different groups. The quantification of the α-subunit of the *K*_Ca_ channels expression was performed by scanning the intensity of the densitometry value (*n* = 5 for each group; values represent the mean ± standard deviation). **(C)** The α-subunit of the *K*_Ca_ channels mRNA expression changes by a qRT-PCR analysis in the tumors from the different groups (*n* = 5 for each group; values represent the mean ± standard deviation). ^*^Indicates a significant difference compared with the control group (*p* < 0.05). ^#^Indicates a significant difference compared with MECUS group (*p* < 0.05).

Immunohistochemistry showed that the protein expression of JAM-A and the α-subunit of the *K*_Ca_ were located mainly in both the capillary endothelium and the tumor cells in glioma (Figures 4A, 5A). In the MEUS groups, the immunohistochemistry, qRT-PCR and Western blot results confirmed that the mRNA and protein expression of JAM-A was decreased compared with the control group, and in the MEIUS group, it decreased more significantly than in the MECUS group (Figure 4). These results demonstrated that MEUS decreased JAM-A expression in glioma, and MEIUS exhibited this more obviously than MECUS. In contrast, the immunohistochemistry, qRT-PCR and Western blot results confirmed that the mRNA and protein expression of the α-subunit of the *K*_Ca_ channels was increased compared with the control group, and the MEIUS group increased more significantly than the MECUS group (Figure 5). These results demonstrated that MEUS increased expression of the α-subunit of the *K*_Ca_ channels in glioma, and MEIUS exhibited this more obviously than MECUS.

### Histologic changes

**Figure 6:**
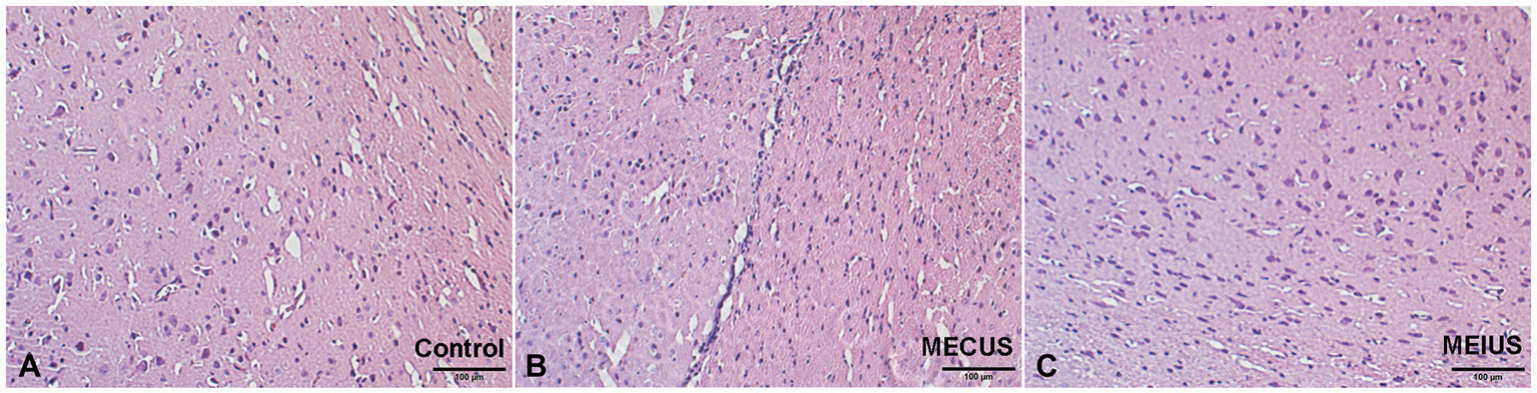
**Hematoxylin-eosin-stained brain samples from the different groups**. A histological analysis showing no relevant differences in the brain tissues from the different groups. **(A)** Control group. **(B)** Microbubble-enhanced continued diagnostic ultrasound group. **(C)** Microbubble-enhanced intermittent diagnostic ultrasound group.

**Figure 7:**
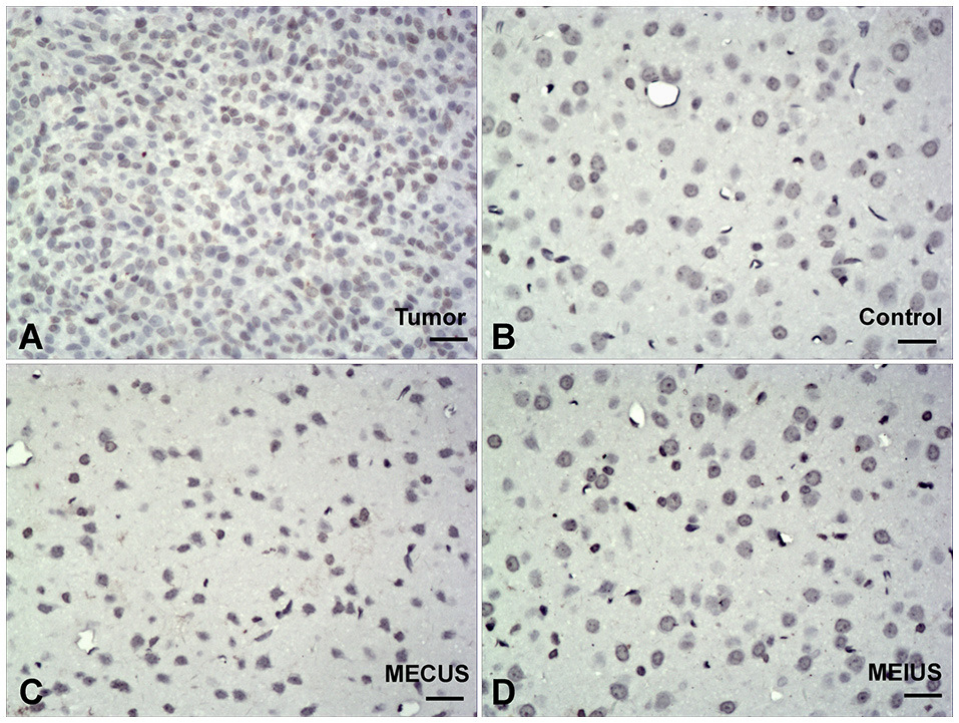
**TUNEL-stained brain samples from the different groups (scale bar = 40 um)**. TUNEL staining showing no apoptosis in the normal brain tissues from the different groups. **(A)** Positive control in tumor. **(B)** Control group. **(C)** Microbubble-enhanced continued diagnostic ultrasound group. **(D)** Microbubble-enhanced intermittent diagnostic ultrasound group.

The Hematoxylin-eosin-staining and TUNEL staining showed that the brain tissues from the different groups did not differ in alterations or microscopic damage. The histological structure was normal, and no hemorrhages, inflammation, apoptosis or other abnormalities were observed (Figures 6, 7). These results demonstrated that MEUS did not cause damage to the normal brain tissue in this study.

## Discussion

Malignant glioma has a high recurrence and mortality rate. The current strategies involve surgical debulking, radiation therapy, and chemotherapy. Drug therapy plays an important role in the treatment of malignant gliomas (Mathieu and Fortin, 2006; Nieder et al., 2009). However, various parts of glioma with a mainly intact BTB may be shielded from many drugs (Black and Ningaraj, 2004). Therefore, there is an urgent need to open the BTB to improve drug therapy for glioma. Recently, the most widely used ultrasound device applied in MEUS is the focused ultrasound therapy apparatus. Therapeutic drugs have been delivered successfully to tumor tissues with focused ultrasound (Diaz et al., 2014; Aryal et al., 2015). However, focused ultrasound usually needs an appropriate image-monitoring system. Therefore, we tried to use diagnostic ultrasound, which is more convenient and economical for mediating microbubble destruction. Low-frequency ultrasound penetrates into tissue easily with few sound energy absorptions and little tissue damage (Xia et al., 2012). Thus, we selected the lowest ultrasound frequency of 1.7 MHz in S5-1 transducer (IE33) in our study in order to go through the skull to increase the BTB permeability. Because the temporal bone is the thinnest bone in the skull, we selected the temporal bone as the ultrasound window. In our preliminary experiment, we found leakage of red blood cells in normal brain tissue when the mechnical index of the ultrasound was 1.4. So the mechnical index of 1.3 was adopted in this study and we found no leakage of red cells. The duration of ultrasound sonication was 10 min according to our previous study (Zhang et al., 2016). The concentration of microbubbles in blood will keep high level within this time.

In this study, we quantitatively and qualitatively investigated the EB deposition in the glioma tissues. EB binds with albumin, the smallest macromolecular component, when injected into blood vessels. It is similar to many small molecular agents that bind effectively to albumin (Saunders et al., 2015). Under normal conditions, the BTB can prevent EB from entering the tumor tissues. However, EB can penetrate into the tumor interstitium when the BTB permeability increases. In our study, we found that the EB extravasation in the MEUS groups exhibited a significant increase compared with the control group (Figure 1). This finding indicates that diagnostic ultrasound, in the presence of microbubbles, induces cavitation, which increases the BTB permeability and causes EB to leak out. The MEIUS group had more EB extravasation than the MECUS group. Other investigators had also shown that intermittent insonification produce more renal hemorrhage than continuous insonification after the administration of microbubbles (Wible et al., 2002). Their results support the observation in our study. With intermittent insonification, microbubbles may circulate undisturbed during the intervals between ultrasound exposures. During this period, the microbubbles have time to flow from larger vessels into the capillary regions. In our study, MEIUS supplied intermittent time (2 s) for microbubble reperfusion after acoustic cavitation and offered more cavitation nuclei to activate than MECUS. In the control group, there was also deposition of EB in the tumor interstitium. The probable reason is that the blood vessels in glioma do not have a fully intact BBB and are somewhat permeable.

In this study, we also adopted the *K*_trans_ of a DCE-MRI to measure the BTB permeability changes in glioma. DCE-MRI is used to evaluate microvascular permeability and drug delivery efficiency after BBB disruption in the brain (Park et al., 2012; Thompson et al., 2013). In our study, the results obtained using the *K*_trans_ values to evaluate BTB permeability were similar to the results of the EB extravasation changes. In addition, the *K*_trans_ values correlated strongly with the EB extravasation in the tumor. This result was consistent with a previous study (Yang et al., 2014), which adopted a Tofts-Kermode model. In our study, we applied diagnostic ultrasound instead of focused ultrasound. However, the *K*_trans_ maps showed that there were no obvious changes as tumor tissue in the surrounding brain tissue after MEUS. The reasons might be as follows: First, glioma has a highly angiogenic vascularization, and thus there was more microbubble accumulation in the tumor than in the normal brain tissue. Furthermore, the glioma vasculature is defective and vulnerable, which becomes a sensitive target for acoustic cavitation. Second, the *K*_trans_ maps did not have enough sensitivity to distinguish minimal changes in the BBB permeability of the surrounding normal brain tissue.

In our study, we investigated the mechanism by which the BTB permeability is increased by MEUS. Some studies show that ultrasound irradiation, combined with microbubbles, opens the BBB by paracellular and transcellular pathways. The integral tight junction proteins are claudins, occludin and junctional adhesion molecules (JAMs). Studies demonstrate that ultrasound combined with microbubbles cause expression changes of tight junction proteins, such as claudin-5, ZO-1 and occluding, in the endothelial cell membrane of the BBB (Sheikov et al., 2008). JAM-A is implicated in a variety of physiologic and pathologic processes involving cellular adhesion, such as tight junction assembly (Yeung et al., 2008). In this study, we found that MEUS decreased JAM-A expression in glioma. Thus, we can conclude the down-regulation of JAM-A expression in glioma affects the formation of tight junctions in the BTB, and this might be one reason for the increasing BTB permeability that is induced by MEUS. *K*_Ca_ channels are symbolic proteins and play an important role in the transcellular pathway regulation of the BTB permeability. These proteins are mainly responsible for the transcellular vesicular transport of the macromolecular through the brain endothelial cell protoplasmic membrane (Ningaraj et al., 2003). In this study, we found that MEUS increased the *K*_Ca_ channels expression in glioma. Thus, we can conclude that the up-regulation of the *K*_Ca_ channels expression in glioma to affect transcellular pathway of crossing BTB might be one reason for the increasing BTB permeability induced by MEUS.

In this study, since the diagnostic ultrasound provides 2-D plane energy scanning, the normal brain tissue could also affected by the scanned diagnostic ultrasound exposure. However, we did not find microscopic damage in the normal brain tissue. The probable reason might be that the diagnostic ultrasound in this study had less output (MI = 1.3). Meanwhile, the glioma vasculature is defective and vulnerable, making it more sensitive than normal brain capillaries to acoustic cavitation. Therefore, we found that MEUS increased the BTB permeability in glioma, and we did not find microscopic damage to the normal brain tissue.

Despite these findings, this study has many limitations. For example, we did not precisely measure the ultrasound power after it went through the skull. Additionally, we did not evaluate the long-term histological effect of the MEUS treatment on the brain.

In summary, MEUS increased the BTB permeability in a rat glioma model without causing damage to the normal brain tissue, at least, immediately after the procedure. The mechanism might involve affecting formation of tight junctions by reducing JAM-A expression and promoting pinocytosis by increasing *K*_Ca_ channels expression in glioma. Additionally, the *K*_trans_ value could be a non-invasive method to evaluate the BTB permeability in rat glioma after a microbubble-enhanced ultrasound treatment. These findings might provide some new guidance for glioma drug therapy.

## Author contributions

Conceived and designed the experiments: WZ, ZL, and JZ. Performed the experiments and acquired data: JZ, HL, XD, YG, XC, JF, and SW. Analyzed the data: JZ, HL, XD, and PC. Contributed reagents/materials/analysis tools: JZ, PC, and BZ. Wrote the paper: JZ, WZ, and ZL.

## Funding

This work was partially supported by the National Natural Science Foundation of China (Grant No. 81571660) and Chongqing Science and Technology R & D Base Construction (International Cooperation) Project (No. cstc2014gjhz110002).

### Conflict of interest statement

The authors declare that the research was conducted in the absence of any commercial or financial relationships that could be construed as a potential conflict of interest.

## Abbreviations

BTB: blood-brain tumor barrier
MEUS: microbubble-enhanced diagnostic ultrasound
MEIUS: microbubble-enhanced intermittent diagnostic ultrasound
MECUS: microbubble-enhanced continued diagnostic ultrasound
DCE-MRI: dynamic contrast-enhanced-MRI.
